# Evaluation of a Genetics Education Program for Health Interpreters: A Pilot Study

**DOI:** 10.3389/fgene.2021.771892

**Published:** 2022-02-03

**Authors:** Miranda E. Vidgen, Lindsay F. Fowles, Satrio Nindyo Istiko, Erin Evans, Katrina Cutler, Kate Sullivan, Jessica Bean, Louise Healy, Gary Hondow, Aideen M. McInerney-Leo, Gregory Pratt, Deborah Robins, Stephanie Best, Keri Finlay, Priya Ramarao-Milne, Nicola Waddell

**Affiliations:** ^1^ QIMR Berghofer Medical Research Institute, Brisbane, QLD, Australia; ^2^ Genetic Health Queensland, Royal Brisbane and Women’s Hospital, Brisbane, QLD, Australia; ^3^ Queensland Genomics Community Advisory Group, The University of Queensland, Brisbane, QLD, Australia; ^4^ Health Consumers Queensland, Brisbane, QLD, Australia; ^5^ Queensland Genomics, The University of Queensland, Brisbane, QLD, Australia; ^6^ Dermatology Research Centre, The University of Queensland Diamantina Institute, The University of Queensland, Brisbane, Brisbane, QLD, Australia; ^7^ Australian Genomics, Murdoch Children’s Research Institute, Melbourne, VIC, Australia; ^8^ Macquarie University, Sydney, NSW, Australia; ^9^ Genetic Support Network of Victoria, Melbourne, VIC, Australia; ^10^ Australian e-Health Research Centre, Health and Biosecurity, CSIRO, Brisbane, QLD, Australia

**Keywords:** genomics, genetics, education, medical interpreter, health interpreter, culturally and linguistically diverse, implementation, evaluation

## Abstract

Health Interpreters enable effective communication between health practitioners and patients with limited knowledge of the predominant language. This study developed and evaluated a training session introducing Health Interpreters to genetics. The online training was delivered multiple times as a single 2-h session comprising lectures and activities. Participants completed questionnaires (pre-, post-, and 6-months follow-up) to assess the impact of training on knowledge, attitude, self-efficacy, and self-reported practice behaviour. Questionnaires were analysed using descriptive statistics, Fisher’s Exact, or independent *t*-test. In total, 118 interpreters participated in the training sessions. Respondent knowledge improved, with gains maintained at 6-months (*p* < 0.01). There were no changes in self-efficacy, and attitudes. Training did not change self-reported practice behaviour, but there was notable pre-existing variability in participants’ methods of managing unknown genetic words. Most respondents agreed that training was useful (93%) and relevant (79%) to their work. More respondents reported learning more from the case study activity (86%) than the group activity (58%). Health Interpreters found the training acceptable and demonstrated sustained improvement in knowledge of genetic concepts. Increased delivery of this training and associated research is needed to assess findings in a larger cohort and to measure the impact on patients.

## Introduction

Health Interpreters provide a vital service within health systems for patients with limited knowledge of the predominant local language. Their involvement in clinical care is associated with improved quality of healthcare (Karliner et al., 2007). Relaying clinical information accurately to patients is a well-known barrier to effective clinical care, even to native speakers (Meuter et al., 2015). Patients with limited proficiency in the local language may experience further barriers, especially in a situation with technical terminology or high stress (Booth and Tickle, 2003; Cohen et al., 2005). In Australia, 22.2% of households speak one of over 300 languages-other-than-English (LOTE), including sign-languages (Australian Bureau of Statistics, 2016). Mandarin (2.5%), Arabic (1.4%), Cantonese, Vietnamese and Italian (1.2%) are the most common LOTE (Australian Bureau of Statistics, 2016). Since 2008, Australia’s public health services have provided Health Interpreters, free of charge, for patients with limited English at the request of the patient or clinician (Australian Commission on Safety and Quality in Healthcare, 2008). Clinicians are discouraged from using non-professional interpreters (e.g. family members) (Queensland Health Interpreter Service, 2007), as use of non-professional interpreters in health settings is associated with poorer clinical outcomes (White et al., 2018).

Over the last decade genomic testing in clinical care has been increasing (Gaff et al., 2017; Burns et al., 2019; Stark et al., 2019; Vidgen et al., 2021). Training in genetic and genomic terminology for Health Interpreters and non-specialist interpreters who work in medical settings has been identified as an unmet area of need to improve patient outcomes (Krieger et al., 2018; Lara-Otero et al., 2019; Uebergang et al., 2021). This was supported by anecdotal reports of challenges in working with health interpreters from clinicians within the local genetics service. In Australia, interpreters of common LOTE working in medical settings have additional qualifications, with training delivered in the LOTE and a qualification as a certified specialist health interpreter (National Accreditation Authority for Translators and Interpreters, 2020a). However, interpreters working with a LOTE with limited diffusion in the community do not have access to additional language-specific health interpreter training. These interpreters work as paraprofessionals as either certified provisional or recognized practising interpreters (National Accreditation Authority for Translators and Interpreters, 2020a). Language skills in specialist areas of medicine come from work-based practice or post-certification professional development.

Post-qualification training of non-genetic health professionals in genetic and genomic concepts is common for physicians, nurses, and allied health professionals (Talwar et al., 2017). This approach in professional upskilling in genetics and genomics has been effective in improving knowledge and, in some cases, has been demonstrated to positively impact clinical practice (Blazer et al., 2004; Carroll et al., 2009; Metcalf et al., 2010). These finding suggest a similar approach could benefit other trained professionals including interpreters. While internationally there are examples of health interpreter training in prenatal and paediatric genetic terminology (Roath et al., 2019; Roath et al., 2020), we could not identify any examples of general genetics training available to Australia’s Health Interpreters. Interpreters are required to participate in professional development activities, including short courses, as part of their certification for continued practice in Australia (National Accreditation Authority for Translators and Interpreters, 2020b). However, there is little research into the impact of short courses and one-off training sessions on their professional development.

Here we describe an interactive training session aimed to introduce Health Interpreters to basic genetic and genomic concepts and their clinical application. This study’s objective was to evaluate the training sessions’ effectiveness in improving Health Interpreters’ knowledge, attitude, confidence, and practice behaviour using genetic and genomic terms in their professional practice.

## Materials and Methods

### Context

**FIGURE 1:**
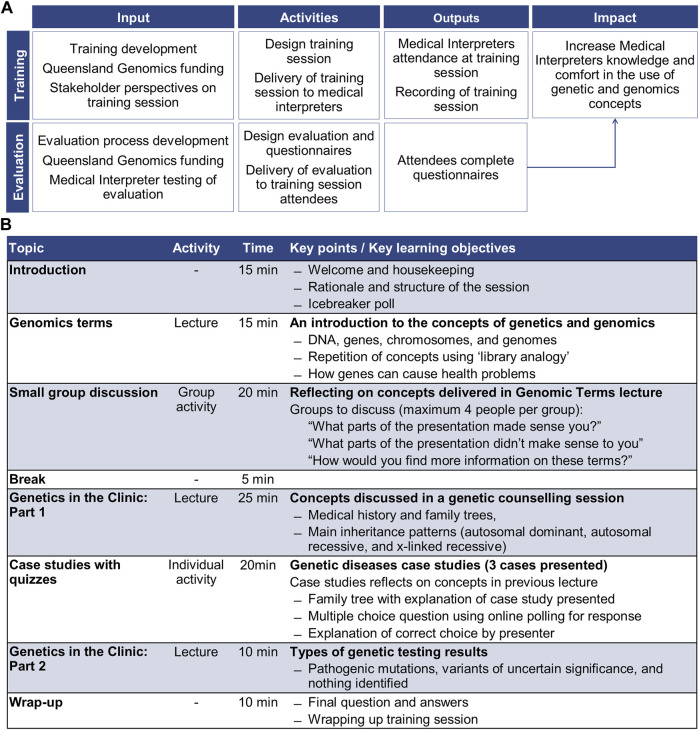
Summary of the training session and evaluation; **(A)** program logic for training session and evaluation, and **(B)** training session structure.

The training involved an interactive workshop-style session, delivered in English to a mixed language cohort of professionally qualified interpreters (see Supplementary Material S1 for health interpreters in the Australian context). It gave Health Interpreters that participated an introduction to key genetic and genomic terms that are applicable to clinical practice. The objective was for participants to be able to recognise genetic and genomic terms, in English, that are commonly discussed in clinical consultations. From the awareness and knowledge gained through the training, participants were encouraged to explore options for interpreting these words in the language(s) they interpret in their own time. The session was delivered three times using the online Zoom meeting platform (Zoom Video Communications, 2020) between July and August 2020. The online platform and method were selected due to local restrictions on in-person meetings caused by COVID-19. The training and associated evaluation was intended to assess changes in Health Interpreters knowledge and comfort in the use of genetic and genomics concepts in their professional practise (Figure 1A). Participants could claim professional development points for attending, which contributes towards continued certification by the National Accreditation Authority for Translators and Interpreters (Australia).

A genetic counsellor (LFF) and a genomic research academic with tertiary teaching experience (MEV) designed the content and format of the training session. The training content was based on the experiences of genetic counsellors working with interpreters in clinical practise. Interpreter service providers were consulted regarding the training session structure and delivery. The 2-h training session was comprised of three lectures and two activities (Figure 1B).

### Participants and Recruitment

Interpreter service providers, which Queensland Health contracts to provide interpreters for the public health service, advertised the training session using promotional materials provided by Queensland Genomics (the sponsors). These providers advertised the training sessions to their contractors through direct email, newsletters, and social media posts.

Training session participants were recruited to the evaluation study *via* an invitation email with a webpage link to both the participant information sheet and the questionnaire. The email for the pre-training session questionnaire was sent to registered participants 1 week prior to the training session. The post-session questionnaire invitation was sent to participants immediately after completing the training sessions and was open for 1-week. The 6-months follow-up questionnaire invitation was sent to participants 6-months after the training session with the questionnaire open for responses for 1-week. Completing the surveys was voluntary and not a prerequisite for attending the training session or receiving professional development points.

### Data Collection and Procedures

The training sessions were evaluated using online questionnaires: pre, post, and 6-months follow-up. The questionnaire applied the Theoretical Domains Framework (Atkins et al., 2017) as the underlying concept to frame questions to investigate participant changes: knowledge, attitudes, self-efficacy, self-seeking behavior for education, and self-reported practice behaviour. The questionnaires were intended to be linked through a self-determined code. Participants were asked to create a 7-character code using; first three letters of the month they were born, the last two numbers of their phone number, and the last two letters of the city they were born (Supplementary Material S2).

Each of the three questionnaires administered the same core 31-items, with post- and 6-months follow-up having additional questions. The response options for the questions included; rating scale, 5-point Likert scale, multiple-choice, and open text boxes. The core questions contained: 8-items assessing demographic information; 3-items assessing self-efficacy of understanding and interpreting genetic terms; 3-items assessing attitude on the importance of genetic health services to themselves or their family and their professional practice; 7-items evaluating self-assessed practice behaviours when interpreting genetic terms in a clinical appointment; and 10-items assessing knowledge of genetic concepts.

The knowledge questions were from a validated knowledge tool (Fitzgerald-Butt et al., 2016). In this study 10 of the 18-items from the validated knowledge tool were used (item numbers in the original publication: 1, 3, 4, 9, 12, 13, 14, 16, 17, and 18) (Fitzgerald-Butt et al., 2016). Item 16, “Humans have 20 pairs of chromosomes”, was validated as a false statement (Fitzgerald-Butt et al., 2016). A questionnaire tester in this study identified an inability to answer the question since it is true to state that humans have 20 pairs of chromosomes. However, it is false to state that humans have only 20 pairs of chromosomes. The investigators changed the item wording to “humans have 24 pairs of chromosomes” to create an unambiguously false statement.

In addition to the core questionnaire items, the post-questionnaire had an additional 10-items evaluating the participants’ training session experience (total 43-items in the post-questionnaire). The 6-months follow-up questionnaire had an additional 5-items capturing the experience of interpreting genetic concepts in the 6-months since the training session (total 36-items in the 6-months follow-up) (Supplementary Material S2).

Items, other than knowledge questions, were customised for this questionnaire. Before use, the follow-up questionnaire was pilot tested by Health Interpreters (*n* = 11) and all questionnaires were reviewed by content experts (*n* = 3). The follow-up questionnaire contained the same core questions as the pre- and post-questionnaires. Only the questions related to the training evaluation which were specific to the post-questionnaire were not included in the pilot test.

### Data Analysis

Descriptive statistics were used to summarise data characteristics for the questionnaire responses. Association between demographic variables and questions related to the domains self-efficacy, attitude, and self-reported behaviour were compared using Fisher’s Exact test. Mean changes in knowledge between questionnaires were analysed with an independent *t*-test. For the 6-months follow-up questionnaire, comparisons were done for each of the domains between, 1) participants that sought additional education (self-seeking behaviour) and 2) participants that had post-intervention appointments, and those that did not.

For the analysis, the variables were collapsed into two or three categories. The variables age, years working as a Health Interpreter, and language interpreted were collapsed into three categories. The language categories were Asian, European and other languages (included African, Oceanian and Middle-Eastern) with languages categorised based on the region of language origin. For example, Spanish originated in Europe, so it is classified as a European language. Variables related to past training and work experience were reduced to two categories, with “unsure” combined with “no”. Likert scale questions for self-efficacy, attitude, and self-reported behaviour were reduced to two categories. The categories that expressed overall ease, positive attitude, and agreement were combined (e.g. strongly agree and agree), as were those that expressed overall difficulty, negative attitude, and disagreement (e.g. strongly disagree, disagree and undecided). Responses collected in open text fields for self-reported practice behaviour were thematically analysed by manual coding (MEV and PRM), using process previously described (Nowell et al., 2017). Results from the statistical analysis were considered to be significant when *p* ≤ 0.05. Analyses were conducted in Stata (version 15.1) (StataCorp, 2017).

## Results

### Questionnaire Responses

There were 180 registered participants, with 118 participating in the training sessions. The pre-questionnaire was sent to 180 registered participants, 37 started answering the questionnaire (response rate 20.5%), but four were excluded as they were incomplete. There were 33 complete responses to the pre-questionnaire. Of the 118 participants who attended the sessions, 48 (response rate 40.7%) and 24 (response rate 20.3%) started the post and 6-months follow-up questionnaires, respectively. After excluding incomplete responses, 43 post responses and 22 6-months follow-up responses were included in the analysis. Of the respondents, six completed all three questionnaires as identified *via* the self-determined code. Given the very low sample size of linked data (*n* = 6), paired analysis suited to longitudinal datasets was not possible due to a lack of power in the analysis. Unpaired statistical methods were used for the analysis of this data.

### Training Session Participant and Questionnaire Respondent Demographics

Training session participants (*n* = 118) interpreted 49 spoken languages, 26 of these languages were interpreted by one participant. No sign language interpreters attended. The majority of training session participants interpreted Asian languages (59.8%), with Mandarin (24%), Vietnamese (13%), Arabic (6%), and Korean (6%) being the most common. Interpreter languages (by region) were similar between training session participants and the questionnaire respondents (Table 1).

**TABLE 1:** Socio-demographic characteristics and professional experience of questionnaire respondents, and languages interpreted by training session participants.

Demographic variables	Training session participants N (%)	Questionnaire respondents		
		Pre N (%)	Post N (%)	6-months follow-up N (%)
Age		N = 33	N = 43	N = 22
25–44	—	10 (30.4)	13 (30.2)	7 (31.8)
45–64	—	19 (57.6)	21 (48.8)	8 (36.3)
65 plus	—	4 (12.1)	9 (20.9)	7 (31.8)
**Gender**		**N = 33**	**N = 43**	**N = 22**
Female	—	30 (90.9)	39 (90.7)	16 (72.7)
**Number of years working as a Health Interpreter**		**N = 33**	**N = 43**	**N = 22**
Not a Health Interpreter	—	5 (15.2)	3 (7.0)	3 (13.6)
0–5 years	—	10 (30.3)	13 (30.2)	8 (36.3)
6 years or more	—	18 (54.5)	27 (62.8)	11 (50.0)
**Before the training session, did you have any training in genetics?**		**N = 33**	**N = 43**	**N = 22**
None at all	—	19 (57.6)	25 (58.1)	17 (77.3)
Some in high school or university	—	10 (30.3)	13 (30.2)	3 (13.6)
Professional development or continued education	—	4 (12.1)	5 (11.6)	2 (9.1)
**What language(s) are you qualified to interpret?** ^†^	**N = 122**	**N = 34**	**N = 4**4	N = 23
Asian language	73 (59.8)	18 (52.9)	25 (56.8)	14 (60.9)
European language	23 (18.9)	8 (23.5)	10 (22.7)	5 (21.7)
Other	25 (20.5)	7 (20.9)	9 (20.5)	4 (17.3)
African language	7 (5.7)	4 (11.8)	4 (9.1)	1 (4.3)
Middle-Eastern language	15 (12.3)	2 (5.9)	4 (9.1)	3 (13.0)
Oceanian language	3 (2.5)	1 (2.9)	1 (2.3)	0
No response	1 (0.8)	1 (2.9)	0	0
**Professional experience**		**N = 33**	**N = 43**	**N = 22**
Have you interpreted for a specialist genetic clinician (clinical geneticist or genetic counsellor)?—Yes	—	9 (27.3)	12 (27.9)	7 (31.8)
Have you interpreted genetic or genomic terms for a health service client before who was not a specialist genetic clinician (clinical geneticist or genetic counsellor)?—Yes	—	4 (12.1)	13 (30.2)	6 (27.3)
Have you had personal experience outside your professional role (e.g. you, a friend or family member) with a serious genetic condition?—Yes	—	6 (18.2)	15 (34.9)	5 (22.7)
Since completing the training session, have you had a client appointment where you interpreted genetics terms?—Yes	—	—	—	4 (18.2)
**Since completing the training session, have you participated in any additional learning about genetics and genomics?**				**N = 22**
Yes	—	—	—	13 (59.1)
Materials provided from the training session	—	—	—	8 (36.4)
Other materials not provided in the training session	—	—	—	1 (4.5)
Both materials provided from the training session and materials not provided in the training session	—	—	—	4 (18.2)
**Since completing the training session, in how many appointments have you interpreted genetic terms?**				**N = 4**
1 to 3	—	—	—	2 (50.0)
4 to 6	—	—	—	1 (25.0)
6 or more	—	—	—	1 (25.0)

† Some training session attendees and questionnaire respondents interpreted for multiple languages from multiple regions. The percentage is based on the number of languages by region spoken by participants, not the number of participants.

There was no statistical difference between demographic variables of the questionnaire respondents across the three response points (Table 1 and Supplementary Material S3, Supplementary Table S1). Respondents tended to be women (pre: 90.9%; post: 90.7%; 6-months follow-up: 72.7%) with more than 6 years interpreting experience (pre: 54.5%; post: 62.8%; 6-months follow-up: 50%), and without educational experience of genetics or genomics (pre: 57.6%; post: 57.1%; 6-months follow-up: 77.3%) (Table 1). Over a quarter of respondents had previously interpreted for genetic health services (pre: 27.3%, post: 27.9%; 6-months follow-up: 31.8%).

### Between Questionnaire Analysis to Assess Changes Over Time

**FIGURE 2:**
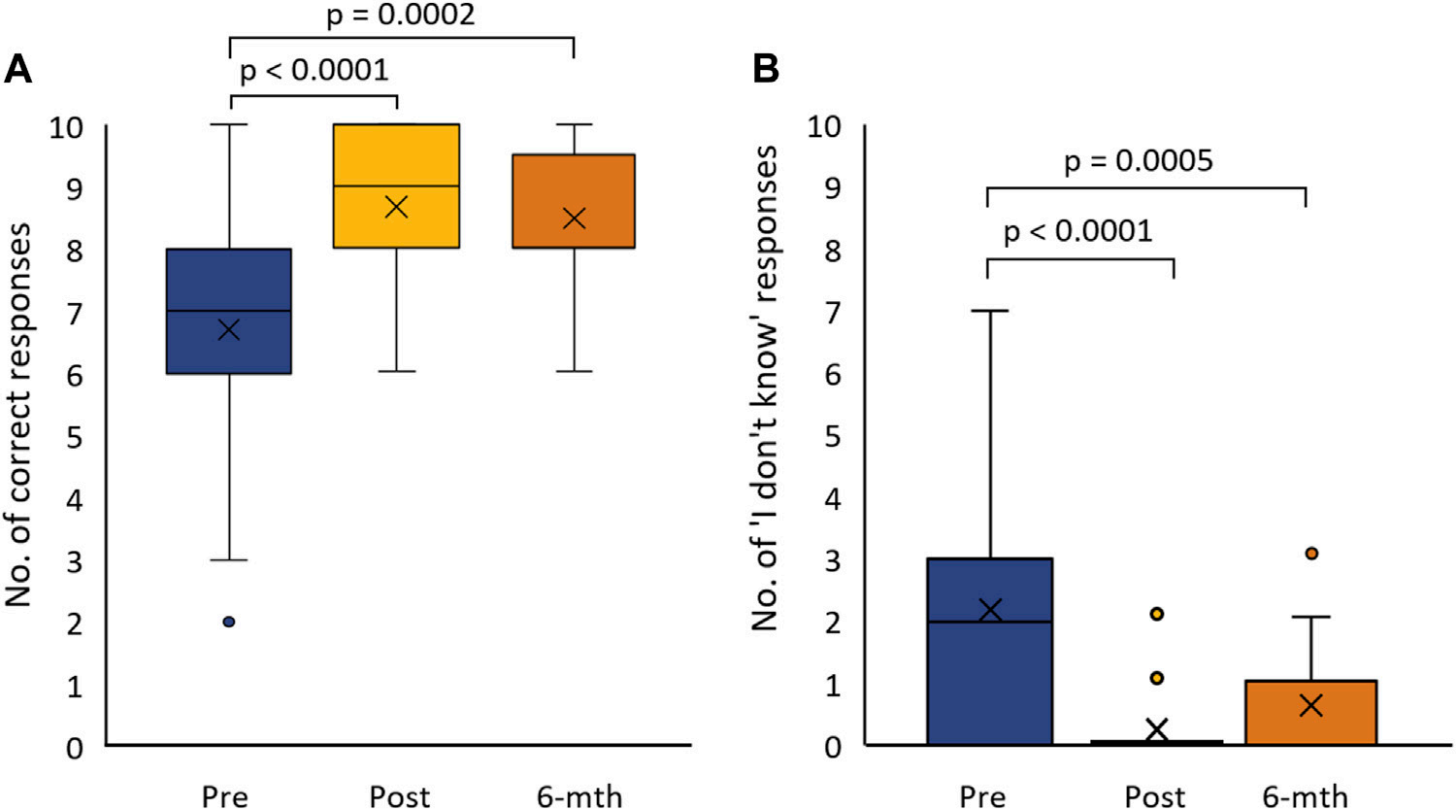
Box-plot of questionnaire respondent knowledge pre-post and 6-months follow-up from the training session: **(A)** the number of correct responses to knowledge questions (Total 10 questions), and **(B)** the number of times respondents selected the ‘I do not know’ response option for knowledge questions.

Knowledge of basic and applied genetic concepts improved significantly after the intervention (pre mean = 6.7, post mean = 8.7; pre-post *t*-test *p* < 0.0001) and remained consistent in the 6-months follow-up (6-months follow-up mean = 8.5; pre-post *t*-test *p* = 0.0002) (Figure 2A). Compared to the pre questionnaire, the “I do not know” response rate significantly reduced after the training session in the post (pre mean = 1.5, post mean = 0.2; pre-post *t*-test *p* < 0.0001) and 6-months follow up questionaries (pre mean = 1.5, 6-months follow-up mean = 0.6; pre-post *t*-test *p* = 0.0005) (Figure 2B). While there was an increase in this response option between post and 6-months follow-up, the change did not reflect a change in overall knowledge. There was no statistically significant difference in self-efficacy or attitude (Supplementary Material S3, Supplementary Table S2).

**FIGURE 3:**
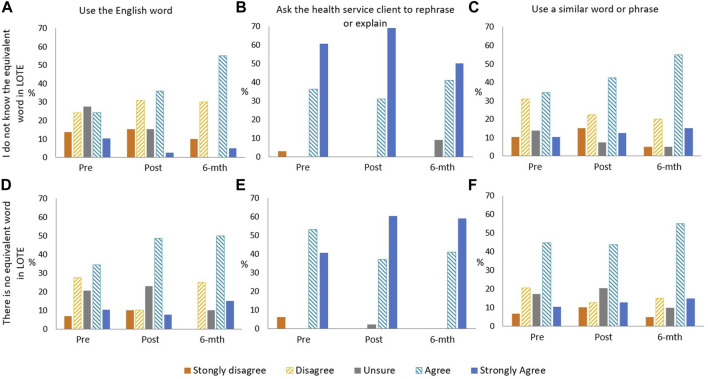
Bar graphs of questionnaire respondent level of agreement of practice behaviours when: they do not know the English word used: **(A)** use the English word, **(B)** ask health service client to rephrase or explain, **(C)** use similar word or phrase; and there is no equivalent word in LOTE, **(D)** use the English word, **(E)** ask health service client to rephrase or explain, **(F)** use a similar word or phrase.

There was no change in self-reported behaviour after attending a training session. “Asking the clinician to rephrase or explain” was the most common action for “words not known by the interpreter” and “words that do not have an equivalent in LOTE”, for both multiple-choice (Figures 3B & E) and open response questions (Table 2). The overall agreeability of using an English word or interpreter selected explanation of terms has a bimodal distribution pattern (Figures 3A,D,E &F). Some respondents provided a mix of options for managing unknown words or words without an equivalent in LOTE in the open response question. Others indicated that asking the clinician to clarify was their only acceptable strategy, using terms such as “mouthpiece” and “conduit” to emphasise the clinician’s responsibility for judgment and explanations.

**TABLE 2:** Thematic summary of open response questions related to practice behaviour.

Themes	Codes	Pre N (%)	Post N (%)	6-months follow-up N (%)
		N = 36^a^	N = 49^a^	N = 23^a^
Ask clinician for clarification^‡^	• Simplify or use layman terms	12 (36.4)	23 (53.5)	8 (36.4)
	• Use different terms			
	• Use examples			
The Health Interpreter chose LOTE alternative^b^	• Use simplified terms	8 (24.2)	7 (16.3)	4 (18.2)
	• Give extended description			
Use imagery	• Drawings	3 (9.1)	5 (11.6)	3 (13.6)
	• Pictures/images			
	• Scans			
Health Interpreter look-up	• LOTE word	3 (9.1)	2 (4.7)	0
	• Information source for patient			
Client resources from clinician	• Write down keywords in English for patient’s reference	3 (9.1)	2 (4.7)	3 (13.6)
	• Ask for written materials on the patient’s behalf			
Use English word^b^	• Use English word	2 (6.1)	2 (4.7)	2 (9.1)
Use physical or verbal indicators	• Body language	2 (6.1)	2 (4.7)	1 (4.5)
	• Sign language^c^			
	• Change speed or tone of speech			
Repeat back	• Get patient to explain understanding back to the health professional	1 (3.0)	4 (9.3)	1 (4.5)
	• Prompt patient to ask clarifying questions of the health professional			
Interpreter self-education	• Speak to the health professional before the appointment	1 (6.1)	2 (4.7)	2 (4.5)
	• Prior or post-self-learning			
		**N = 28** **^d^**	**N = 38** **^d^**	**N = 18** **^d^**
Multiple themes	• Provided multiple options selected based on circumstances	6 (21.4)	11 (28.9)	5 (27.8)
	• Provided two or more themes done in tandem			

a Number of coded responses.

b Option given in set response questions.

c All questionnaire respondents interpret for spoken languages.

d Number of respondents that responded to the question.

### Post-Intervention Self-Seeking Behaviour (Education) and Professional Experience Analysis

In the 6-months after the training session, over half of the respondents sought additional learning on genetics, either from materials provided by the training session or through other sources (*n* = 13, 59.1%) (Table 1). However, there was no statistical significance for knowledge, self-efficacy, attitude, or self-reported behaviour between those who exhibited self-seeking behaviour (education) and those who did not.

Those respondents that had client appointments in the 6-months follow-up period (*n* = 4) identified having appointments with specialists in; genetics (1 appointment, *n* = 2), allergies and immunology (1 appointment, *n* = 1), breast and endocrine surgery (1 appointment, *n* = 1), gynaecology (2 appointments, *n* = 1), maternity and neonatal medicine (2 appointments, *n* = 1), and paediatrics (3 or more appointments, *n* = 1). No appointments were identified for general practice, or with specialists in cardiology, endocrinology, neurology, oncology, or nephrology. Most appointments where respondents interpreted genetic or genomic terms utilised telehealth or telephone communication. Due to COVID-19 restrictions on in-person appointments during the 6-months follow-up period, this may not represent the usual interpreter experience. Those respondents that had appointments after the training session where they used genetics terms considered it easier to understand genetic and genomic terms in English than those respondents that did not have appointments after the training session (Appointments = 75.0%; No appointments = 23.5%, *p* = 0.088) (Supplementary Material S3, Supplementary Table S2).

### Program Evaluation

**FIGURE 4:**
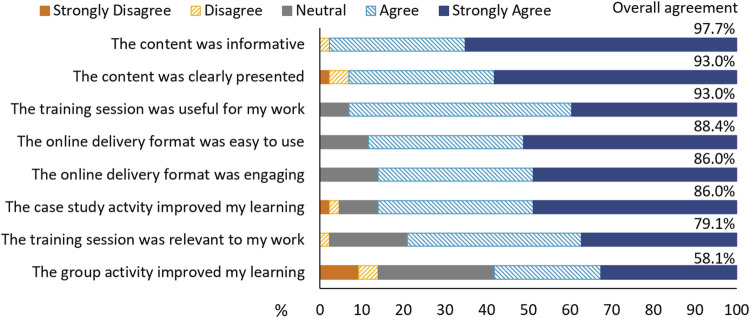
Respondent perspectives of the training session. “Overall agreement” is provided as a percentage and is the combined percentage of “agree” and “strongly agree”.

Respondents had high levels of overall agreement that the training session was clearly presented (93.0%) and informative (97.7%), with it being useful to their work (93.0%), with slightly fewer respondents considering it relevant to their work (79.1%) (Figure 4). Respondents felt that case studies within the training session improved learning (86.0%) more than the group activity (58.1%) (Figure 4). The difference was similarly reflected in the open responses. The use of case studies and quizzes were the most popular activities in the open response questions as they allowed respondents to reflect on the content of the presentations. The use of group discussions was not as well-received due to technical execution using the online platform, in particular communication methods in and between rooms, and the topic discussed (Figure 1). Respondents indicated that they would prefer group discussion on interpreter experiences and expectations during genetic consultation, or role-plays as alternative learning techniques.

Respondents felt the training session could be improved by delivering the content at a slower pace or over multiple sessions. Respondents identified that providing information or slides for the presentations before the training session would assist with participant expectations and support participant learning of complex topics. Respondents also indicated that they would like more examples of different diseases and more details on clinical interactions with genomics (Table 3).

**TABLE 3:** Recommendations for developing training in genetic concepts for Health Interpreters.

Recommendation	Description
Audience background	Health Interpreters do not necessarily come from a scientific or medical background. Educators should not assume prior knowledge. More than 50% of questionnaire respondents had no prior genetics education. Up to 15% interpreted languages of limited diffusion and did not have specific Health Interpreter qualifications
Pace of delivery	Multiple short sessions covering a single topic over weeks or months were suggested by respondents as a preferred pace of delivery to help with the information uptake
Resources	Use a flipped classroom format by providing resources before training (i.e., presentation slides). These can assist participants during sessions delivered in real-time or used as a reference point later
Clinical interaction examples	Information on what they can expect from clinical interactions that involve genetics was a desired inclusion for respondents. This content could be in the context of genetic health service appointments and other specialties. Although in this study, most respondents’ appointments, where genetics was encountered during 6-months follow-up, were with non-genetic medical specialties
Disease-based examples	Reinforcing concepts by health condition examples was preferred
Family context	Providing content in a way that engages Health Interpreters to think about genetics in the context of their own family. Participants engaged with content when explained in the context of their own family
Activities	Respondents felt the presenter walkthrough of case studies and the associated use of quizzes was beneficial to their learning. Other activity styles suggested by respondents were role-play and group discussion of case studies or clinical scenarios
Analogies	Respondents indicated that the use of analogies, such as comparing the human genome to a library, was effective in supporting their learning

## Discussion

Health Interpreters are an essential part of the equitable provision of quality healthcare to people with limited English language skills (Karliner et al., 2007). The use of Health Interpreters is associated with a range of improvements in clinical care for patients with limited English, including; improved clinical outcomes for patients, increased rate of access to health care, decreased admissions and improved patient satisfaction (Jacobs et al., 2001; Bernstein et al., 2002; Karliner et al., 2007).

Continuing professional development of health professionals, including the use of short educational interventions, is a common mechanisms for upskilling health workers (Samuel et al., 2021). The outcome metrics used to measure the success of professional development in healthcare vary considerably between studies depending on the theoretical domains applied to assess change, but changes in knowledge, practise behaviour or patient outcomes are often the focus (Samuel et al., 2021). These types of educational interventions are similarly used in the professional upskilling of Health Interpreters.

Genomics is being implemented across health services with increasing frequency, creating more medical appointments where these complex concepts are discussed. To improve patient outcomes, training in genetic and genomic terminology for Health Interpreters has been identified as an unmet area of need (Krieger et al., 2018; Lara-Otero et al., 2019; Uebergang et al., 2021). While there are some programs for Health Interpreter training in genetic sub-specialties (Roath et al., 2019; Roath et al., 2020), at the time of publication, there hasn’t been evaluation of their effectiveness.

In other studies assessing educational interventions in genetics training for healthcare workers, improved respondent knowledge is consistently observed despite variations in the mode of delivery and duration (Talwar et al., 2017). This was similarly observed in our evaluation of a genetics training session for Health Interpreters, with respondents’ knowledge increasing and being maintained 6-months after the training session. Maintenance of knowledge after an educational intervention does not always occur (Kempegowda et al., 2018). Although the use of “I do not know” response to knowledge questions increased between the post and 6-months follow-up questionnaires, it did not correspond to a change in overall knowledge. However, it may indicate waning confidence in responding to knowledge-based questions. Educational interventions that involve 6-months follow-up, either by formal or self-directed learning, or where participants have professional experience during the 6-months follow-up period further improve knowledge retention and duration of knowledge retention (Masny et al., 2003; Lauer et al., 2014), which reflects adult education principles (McNeil et al., 2006).

The questionnaire assessed self-efficacy and attitude to self/family and professional practice in the context of genetics and genomics. There was no change in either domain observed in this study. Self-efficacy is context-specific within individuals, with day-to-day circumstances influencing perception and confidence about professional practice (Zamani-Alavijeh et al., 2019). The neutrality and lack of change in self-efficacy in professional practice could be attributed to situational variability encountered with the core task of interpreting complex terms between the clinical practitioner and non-English speaking clients.

Attitudes to the use of genetics in clinical settings by non-genetic health professionals are dependent on multiple factors including: their clinical specialty, clinical utility of testing in case management, and clinician’s consideration of the individual patient’s needs (Carroll et al., 2009; Paul et al., 2018). The Health Interpreters’ unchanging neutral attitude to genetics in professional practice could reflect the low number of appointments where they use genetics and genomics terminology. In responses to the training session experience, we saw very high overall agreement that the training session was “useful” (93.0%). However, fewer people agreed that it was “relevant” to their work (79.1%). Health Interpreters do not specialise in specific disciplines, rather they work across all areas of medicine. Therefore, it may take time to come across appointments that use this newly acquired knowledge and change attitudes towards relevance to their work. It is also possible that Health Interpreter attitudes will not change, as this group may perceive all medical terms to be equally important to their professional practice.

The presenters did note that participant questions during the training session often referenced their personal history and/or their family members’ histories rather than their professional practice or clients. This indicates that a number of participants found personal relevance in training by connecting genetic concepts and health services utility to their own family. Incorporating personal family elements may be a way of engaging participants in educational interventions in genetic and genomic concepts.

As is experienced with medical language in general, the English terminology for genetics and genomic concept often do not have equivalent words in LOTE. It is part of an interpreters’ professional practise to manage how terms are delivered, for example through the selection of equivalent words or requesting clarification by the speaker. When the interpreter is unaware of an equivalent word, or it does not occur in the LOTE, asking the clinical client for clarification is the preferred method identified by participants in this study. This practice reflects the Australian training practices and the interpreters’ professional code of ethics (Australian Institute of Interpreters and Translators, 2012). There was a bimodal distribution pattern in the responses to self-reported practice behaviour for using the English word and selecting LOTE equivalent - indicating that individual interpreters have different practice behaviour or perspectives. This practice reflects discourse analysis studies of interpreters in health settings that demonstrate how altering messages varies between interpreters (Gutierrez et al., 2017; Gutierrez et al., 2019). These differences in interpreting practice do not appear to be associated with language or culture but rather the individual (Krieger et al., 2018; Lara-Otero et al., 2019). They may be linked to broader concepts, such as the school of thought associated with initial interpreter training and the individual’s past professional experience. Equally, this may reflect individual clinical appointment differences, such as cultural compatibility or the rapport established between the interpreter and the non-English speaking client, rapport with the clinician, or confidence in the interpreted medical topic.

### Future Education Recommendations

There is a lack of professional development opportunities for Health Interpreters in Australia and so there was strong support for this training, with requests from Health Interpreters to increase the scope of training to include other medical specialties. To meet the demand for training, the delivery format may need to be adjusted as using live online sessions may not be practical. This mode restricts availability for some users due to the inflexible timing of sessions, has high resource requirements for delivery, and requiring participants to have a reliable internet connection and an interruption-free environment. Other studies of genetic education have demonstrated effective knowledge increase in non-genetic health professionals when using self-directed online training programs and on-demand recorded sessions tailored to the needs of the specific health profession (Wallen et al., 2011; Kaur et al., 2019). The impact of these other learning formats is unknown for Health Interpreters and would require further investigation if applied to their profession. Here we outline some considerations for developing training in genetic concepts for Health Interpreters based on the presenters’ experiences and participant feedback (Table 3). Whilst technically challenging, future evaluation studies should aim to explore the impact of Health Interpreter educations not only on participants, but also on outcomes for patients and clinical services.

### Limitations

The original design of this research was a linked longitudinal study. The main limitation was the insufficient amount of paired data for analysis, which necessitated unpaired methods for analysis. The analysis type weakens both the power and the longitudinal inference of the results. There were moderate responder rates for each individual survey but very low repeat responder rates. To maintain responder anonymity, we did not collect responder contact details. This meant we could not provide targeted reminders to those who did not complete follow-up surveys and could not provide professional development incentives for participating in the research. The researchers would consider methods for re-contacting participants to improve repeat response rates in the future. There is potential responder bias as only 18–32% of training participants completed each of the questionnaires. Based on the response rate and survey findings we would suggest that the evaluation method and domains explored be reassessed to determine if these metrics are suitable for the assessment training in the context of continuing professional development for Health Interpreters.

## Conclusion

Research has identified that improving Health Interpreters’ knowledge of genetic and genomics concepts would improve their client interactions during genetic counselling sessions (Krieger et al., 2018; Gutierrez et al., 2019; Uebergang et al., 2021). This study demonstrated that short training sessions can be an effective way of improving Health Interpreter knowledge of genetic and genomic concepts relevant to the clinical practice of genetic health services. Here we demonstrate the first step - that the intervention positively impacts a Health Interpreter’s knowledge. The next step in determining the intervention’s value is examining the impact of Health Interpreter training on medical appointments where genetics is discussed from the perspective of the patient, the interpreters, and the clinical staff.

## Data Availability

The data generated by the study will be made available from the corresponding author upon reasonable request.

## References

[B1] Atkins L. Francis J. Islam R. O’Connor D. Patey A. Ivers N. (2017). A Guide to Using the Theoretical Domains Framework of Behaviour Change to Investigate Implementation Problems. Implementation Sci.. Canberra, ACT: Australian Bureau of Statistics 12 (1), 77. 10.1186/s13012-017-0605-9 PMC5480145 28637486

[B2] Australian Bureau of Statistics (2016). 2016 Census QuickStats: Australia. Available at: https://quickstats.censusdata.abs.gov.au/census_services/getproduct/census/2016/quickstat/036 (Accessed February 28, 2020).

[B3] Australian Commission on Safety and Quality in Healthcare (2008). Consumer Guide to Australian Charter of Healthcare Rights. Sydney, NSW: Australian Commission on Safety and Quality in Healthcare. Available at: https://www.safetyandquality.gov.au/our-work/partnering-consumers/australian-charter-healthcare-rights/australian-charter-healthcare-rights-first-edition.

[B4] Australian Institute of Interpreters and Translators (2012). AUSIT Code of Ethics and Code of Conduct. Brisbane, Queensland: Australian Institute of Interpreters and Translators. Available at: https://ausit.org/wp-content/uploads/2020/02/Code_Of_Ethics_Full.pdf.

[B5] Bernstein J. Bernstein E. Dave A. Hardt E. James T. Linden J. (2002). Trained Medical Interpreters in the Emergency Department: Effects on Services, Subsequent Charges, and Follow-Up. J. Immigr Health 4 (4), 171–176. 10.1023/a:1020125425820 16228770

[B6] Blazer K. R. Grant M. Sand S. R. MacDonald D. J. Uman G. C. Weitzel J. N. (2004). Effects of a Cancer Genetics Education Programme on Clinician Knowledge and Practice. J. Med. Genet. 41 (7), 518–522. 10.1136/jmg.2004.018234 15235022 PMC1735845

[B7] Booth H. Tickle L. (2003). The Future Aged: New Projections of Australia. 's Elderly Population. Australas. J Ageing 22 (4), 196–202. 10.1111/j.1741-6612.2003.tb00497.x

[B8] Burns B. L. Bilkey G. A. Coles E. P. Bowman F. L. Beilby J. P. Pachter N. S. (2019). Healthcare System Priorities for Successful Integration of Genomics: An Australian Focus. Front. Public Health 7, 41. 10.3389/fpubh.2019.00041 30915324 PMC6421399

[B9] Carroll J. C. Rideout A. L. Wilson B. J. Allanson J. M. Blaine S. M. Esplen M. J. (2009). Genetic Education for Primary Care Providers: Improving Attitudes, Knowledge, and Confidence. Can. Fam. Physician 55 (12), e92–9. 20008584 PMC2793208

[B10] Cohen A. L. Rivara F. Marcuse E. K. McPhillips H. Davis R. (2005). Are Language Barriers Associated with Serious Medical Events in Hospitalized Pediatric Patients? Pediatrics 116 (3), 575–579. 10.1542/peds.2005-0521 16140695

[B11] Fitzgerald‐Butt S. M. Bodine A. Fry K. M. Ash J. Zaidi A. N. Garg V. (2016). Measuring Genetic Knowledge: a Brief Survey Instrument for Adolescents and Adults. Clin. Genet. 89 (2), 235–243. 10.1111/cge.12618 26032340 PMC5215811

[B12] Gaff C. L. M. Winship I. M. Forrest S. Clark D. M. Waring P. South M. (2017). Preparing for Genomic Medicine: a Real World Demonstration of Health System Change. NPJ Genom Med. 2 (1), 16. 10.1038/s41525-017-0017-4 29263830 PMC5677913

[B13] Gutierrez A. M. Robinson J. O. Statham E. E. Scollon S. Bergstrom K. L. Slashinski M. J. (2017). Portero versus Portador: Spanish Interpretation of Genomic Terminology during Whole Exome Sequencing Results Disclosure. Per Med. 14 (6), 503–514. 10.2217/pme-2017-0040 29749861 PMC6393936

[B14] Gutierrez A. M. Statham E. E. Robinson J. O. Slashinski M. J. Scollon S. Bergstrom K. L. (2019). Agents of Empathy: How Medical Interpreters Bridge Sociocultural Gaps in Genomic Sequencing Disclosures with Spanish-speaking Families. Patient Educ. Couns. 102 (5), 895–901. 10.1016/j.pec.2018.12.012 30581014 PMC7197396

[B15] Jacobs E. A. Lauderdale D. S. Meltzer D. Shorey J. M. Levinson W. Thisted R. A. (2001). Impact of Interpreter Services on Delivery of Health Care to limited-English-proficient Patients. J. Gen. Intern. Med. 16 (7), 468–474. 10.1046/j.1525-1497.2001.016007468.x 11520385 PMC1495243

[B16] Karliner L. S. Jacobs E. A. Chen A. H. Mutha S. (2007). Do professional Interpreters Improve Clinical Care for Patients with Limited English Proficiency? A Systematic Review of the Literature. Health Serv. Res. 42 (2), 727–754. 10.1111/j.1475-6773.2006.00629.x 17362215 PMC1955368

[B17] Kaur R. Meiser B. Zilliacus E. Tim Wong W. K. Woodland L. Watts K. (2019). Evaluation of an Online Communication Skills Training Programme for Oncology Nurses Working with Patients from Minority Backgrounds. Support Care Cancer 27 (5), 1951–1960. 10.1007/s00520-018-4507-4 30327877

[B18] Kempegowda P. Chandan J. S. Hutton R. Brown L. Madden W. Webb J. (2018). Focused Educational Intervention Improves but May Not Sustain Knowledge Regarding Falls Management. BMJ Open Qual. 7 (3), e000222. 10.1136/bmjoq-2017-000222 PMC6059340 30057952

[B19] Krieger M. Agather A. Douglass K. Reiser C. A. Petty E. M. (2018). Working with the Hmong Population in a Genetics Setting: an Interpreter Perspective. J. Genet. Couns. 27 (3), 565–573. 10.1007/s10897-017-0153-0 28942494

[B20] Lara-Otero K. Weil J. Guerra C. Cheng J. K. Y. Youngblom J. Joseph G. (2019). Genetic Counselor and Healthcare Interpreter Perspectives on the Role of Interpreters in Cancer Genetic Counseling. Health Commun. 34 (13), 1608–1618. 10.1080/10410236.2018.1514684 30230379

[B21] Lauer P. A. Christopher D. E. Firpo-Triplett R. Buchting F. (2014). The Impact of Short-Term Professional Development on Participant Outcomes: a Review of the Literature. Prof. Dev. Educ. 40 (2), 207–227. 10.1080/19415257.2013.776619

[B22] Masny A. Daly M. Ross E. Balshem A. Gillespie D. Weil S. (2003). A Training Course for Oncology Nurses in Familial Cancer Risk Assessment: Evaluation of Knowledge and Practice. J. Cancer Educ. 18 (1), 20–25. 10.1207/S15430154JCE1801_10 12825630

[B23] McNeil H. P. McNeil H. P. Hughes C. S. Toohey S. M. Dowton S. B. (2006). An Innovative Outcomes-Based Medical Education Program Built on Adult Learning Principles. Med. Teach. 28 (6), 527–534. 10.1080/01421590600834229 17074700

[B24] Metcalf M. P. Tanner T. B. Buchanan A. (2010). Effectiveness of an Online Curriculum for Medical Students on Genetics, Genetic Testing and Counseling. Med. Ed. Online 15. 10.3402/meo.v15i0.4856 PMC2823389 20174615

[B25] Meuter R. F. I. Gallois C. Segalowitz N. S. Ryder A. G. Hocking J. (2015). Overcoming Language Barriers in Healthcare: A Protocol for Investigating Safe and Effective Communication when Patients or Clinicians Use a Second Language. BMC Health Serv. Res. 15, 371. 10.1186/s12913-015-1024-8 26357948 PMC4566365

[B26] National Accreditation Authority for Translators and Interpreters (2020b). Professional Development Requirements. Available at: https://www.naati.com.au/practitioners/professional-development-requirements/(Accessed March 02, 2021).

[B27] National Accreditation Authority for Translators and Interpreters (2020a). The Certification System. Available at: https://www.naati.com.au/become-certified/(Accessed March 02, 2021).

[B28] Nowell L. S. Norris J. M. White D. E. Moules N. J. (2017). Thematic Analysis:Striving to Meet the Trustworthiness Criteria. Int. J. Qual. Methods 16 (1), 1609406917733847. 10.1177/1609406917733847

[B29] Paul J. L. Leslie H. Trainer A. H. Gaff C. (2018). A Theory-Informed Systematic Review of Clinicians' Genetic Testing Practices. Eur. J. Hum. Genet. 26 (10), 1401–1416. 10.1038/s41431-018-0190-7 29891880 PMC6138746

[B30] Queensland Health Interpreter Service (2007). Working with Interpreters: Guidelines. Brisbane: Queensland Health. Available at: https://www.health.qld.gov.au/__data/assets/pdf_file/0033/155994/guidelines_int.pdf.

[B31] Roath C. E. Aiyar L. Williamson Dean L. Bovee Terry A. Weaver M. (2019). Interpreting for Prenatal Genetics: A Workshop for Interpreters in Health Care. Bethesda, MD: National Coordinating Center for the Regional Genetics Networks. Available at: https://nccrcg.org/wp-content/uploads/2019/02/Interpreting-for-Prenatal-Genetic-Counseling.pdf.

[B32] Roath C. E. Applegate C. Williamson Dean L. Hasegawa-Evans L. Weaver M. (2020). Interpreting for Pediatirc Genetics: A Workshop for Interpreters in Health Care. Bethesda, MD: National Coordinating Centre for teh Regional Genetics Networks. Available at: https://nccrcg.org/wp-content/uploads/2020/02/Interpreting-for-Pediatric-Genetic-Counseling-Curriculum.pdf.

[B33] Samuel A. Cervero R. M. Durning S. J. Maggio L. A. (2021). Effect of Continuing Professional Development on Health Professionals’ Performance and Patient Outcomes: A Scoping Review of Knowledge Syntheses. Acad. Med. 96 (6), 913. 10.1097/ACM.0000000000003899 33332905

[B34] Stark Z. Boughtwood T. Phillips P. Christodoulou J. Hansen D. P. Braithwaite J. (2019). Australian Genomics: A Federated Model for Integrating Genomics into Healthcare. Am. J. Hum. Genet. 105 (1), 7–14. 10.1016/j.ajhg.2019.06.003 31271757 PMC6612707

[B35] StataCorp (2017). Stata Statistical Software. College Station, TX: StataCorp LLC.

[B36] Talwar D. Tseng T. S. Foster M. Xu L. Chen L. S. (2017). Genetics/genomics Education for Nongenetic Health Professionals: a Systematic Literature Review. Genet. Med. 19 (7), 725–732. 10.1038/gim.2016.156 27763635

[B37] Uebergang E. Best S. de Silva M. G. Finlay K. (2021). Communicating Genomic Health Information: Recommendations from Culturally and Linguistically Diverse Communities and Healthcare Interpreters. J. Community Genet. 12 (4), 549–557. 10.1007/s12687-021-00537-0 34185265 PMC8554909

[B38] Vidgen M. E. Williamson D. Cutler K. McCafferty C. Ward R. L. McNeil K. (2021). Queensland Genomics: An Adaptive Approach for Integrating Genomics into a Public Healthcare System. npj Genom. Med. 6 (71). 10.1038/s41525-021-00234-4 PMC8373904 34408148

[B39] Wallen G. R. Cusack G. Parada S. Miller-Davis C. Cartledge T. Yates J. (2011). Evaluating a Hybrid Web-Based Basic Genetics Course for Health Professionals. Nurse Educ. Today 31 (6), 638–642. 10.1016/j.nedt.2010.11.001 21106279 PMC3049200

[B40] White J. Plompen T. Osadnik C. Tao L. Micallef E. Haines T. (2018). The Experience of Interpreter Access and Language Discordant Clinical Encounters in Australian Health Care: a Mixed Methods Exploration. Int. J. Equity Health 17 (1), 151. 10.1186/s12939-018-0865-2 30249270 PMC6154887

[B41] Zamani-Alavijeh F. Araban M. Harandy T. F. Bastami F. Almasian M. (2019). Sources of Health Care Providers’ Self-Efficacy to Deliver Health Education: a Qualitative Study. BMC Med. Educ. 19 (1), 16. 10.1186/s12909-018-1448-z 30626364 PMC6327583

[B42] Zoom Video Communications (2020). Zoom Cloud Meeting". Versions 5.1.3. & 5.2.0. San Jose, CA: Zoom Video Communications, Inc.

