# Supplementary material for: Evaluation of a Genetics Education Program for Health Interpreters: A Pilot Study
Source: Front Genet. 2022 Feb 3;12:771892. doi: 10.3389/fgene.2021.771892 (PMC8850313; doi:10.3389/fgene.2021.771892)
Supplement: Supplementary file 1 [file DataSheet2.PDF]

Table S1: Fisher's Exact analysis of socio-demographic variables to compare pre-, post, and 6-month follow-up questionnaires. Significant p<0.05. Significant tests indicated by * in bold.

	Number categories	Pre N	Post N	6-month follow-up N	p-value		
					Pre vs. Post	Post vs. 6-month follow-up	Pre vs. 6-month follow-up
Age	3	33	43	22	0.578	0.543	0.151
Gender	2	33	43	22	1.000	0.076	0.134
Years working as Health Interpreter	3	33	43	22	0.492	0.494	0.929
Training in genetics	2	33	43	22	1.000	0.173	0.158
Interpreted for a specialist genetic clinician	2	33	43	22	1.000	0.401	0.382
Interpreted genetic or genomic terms for a health service client before who was not a specialist genetic clinician	2	33	43	22	0.095	1.000	0.175
Personal experience outside your professional role (e.g. you, a friend or family member) with a serious genetic condition	2	33	43	22	0.127	0.400	0.739
Language interpreted	3	34	44	23	0.950	1.000	1.000

Table S2: Independent t-test analysis of knowledge and Fisher's Exact analysis of self-efficacy, attitude, and self-reported practice behavior as grouped by questionnaire comparison, self-seeking behavior (education), and appointments post-training. Significant p<0.05. Significant tests indicated by * in bold. Analysis with Independent t-test indicated by [†].

Topic	Question/Item	Questionnaire comparison						Self-seeking (education)			Professional experience		
		Pre	Post	6mth	Pre-Post Sig.	Post-6mth Sig.	Pre-6mth Sig.	Yes	No	Sig.	Yes	No	Sig.
Knowledge	Number of responses from 10 questions	Mean N = 33	Mean N = 43	Mean N = 22				Mean N = 9	Mean N = 13		Mean N = 4	Mean N = 18	
	Number of answers correct [†]	6.7	8.7	8.6	**0.000***	0.555	**0.0002***	8.6	8.4	0.636	8.8	8.4	0.506
	Number of times used 'I don't know' response for questions [†]	2.2	0.2	0.6	**0.000***	0.0504	**0.0005***	0.6	0.6	0.980	0.5	0.6	0.697
Self-efficacy	Rate how easy or difficult you find the following activities. Overall ease combined easy and very easy.	% Overall ease	% Overall ease	% Overall ease				% Overall ease	% Overall ease		% Overall ease	% Overall ease	
	Understanding genetic and genomic terms in English	15.2 (5/33)	25.6 (11/43)	33.3 (7/21)	0.396	0.562	0.180	41.7 (5/12)	22.2 (2/9)	0.642	75.0 (3/4)	23.5 (4/17)	0.088
	Explaining to others how genetics affects a person's health	15.2 (5/33)	18.6 (8/43)	19.0 (4/21)	0.766	1.000	0.723	25.0 (3/12)	11.1 (1/9)	0.603	25.0 (1/4)	17.6 (3/17)	1.000
	Interpreting information about genetics for my clients with limited English, or that are Deaf or hard of hearing	6.3 (2/32)	7.0 (3/43)	23.8 (5.21)	1.000	0.102	0.099	25.0 (3/12)	22.2 (2/9)	1.000	25.0 (1/4)	23.5 (4/17)	1.000
Attitude	Overall positive rating combined more and much more.	% Overall positive rating	% Overall positive rating	% Overall positive rating				% Overall positive rating	% Overall positive rating		% Overall positive rating	% Overall positive rating	
	When compared to other medical services, how would you rate the importance of genetic health services to you and your family?	21.9 (7/32)	48.8 (20/41)	42.1 (8/19)	0.155	0.783	0.389	46.2 (6/13)	33.3 (2/6)	1.000	25.0 (1/4)	46.7 (1/4)	0.582

	When compared to other medical services, how would you rate the importance of genetic health services to your clients with limited English, or that are Deaf or hard of hearing?	40.6 (13/32)	45.0 (18/40)	44.4 (8/18)	0.487	1.000	0.771	50.0 (6/12)	33.3 (2/6)	0.620	50.0 (2/4)	42.9 (6/14)	0.576
	When compared to other medical terminology, how would you rate the importance of genetic and genomic terminology in your professional practice of interpreting	36.7 (11/30)	47.4 (1/38)	35.0 (7/20)	1.000	1.000	0.779	33.3 (3/12)	37.5 (3/8)	1.000	66.7 (2/3)	29.4 (5/17)	0.566
Practice behavior	*In a client appointment, if **I do not know the word/sign** for a genetic term in the language, I am interpreting I…* Overall agreement combined agree and strongly agree.	% Overall agree-ment	% Overall agree-ment	% Overall agree-ment				% Overall agree-ment	% Overall agree-ment		% Overall agree-ment	% Overall agree-ment	
	Use the English word or fingerspell the word	34.5 (10/29)	38.5 (15/39)	60.0 (12/20)	0.803	0.168	0.090	50.0 (6/12)	75.0 (6/8)	0.373	50.0 (2/4)	62.5 (10/16)	1.000
	Ask the health service client to rephrase or explain	97.0 (32/33)	88.1 (37/42)	90.9 (20/22)	0.440	0.115	0.557	84.6 (9/13)	100.0 (9/9)	0.494	100.0 (4/4)	88.9 (16/18)	1.000
	Use a similar term or phrase	44.8 (13/29)	52.5 (21/40)	70.0 (14/20)	0.469	0.402	0.143	66.7 (8/12)	75.0 (6/8)	1.000	50 (2/4)	75.0 (12/16)	0.549
Practice behavior	*In a client appointment, if **there is not an equivalent word/sign** for a genetic term in the language, I am interpreting I….* Overall agreement combined agree and strongly agree.	% Overall agree-ment	% Overall agreeme nt	% Overall agree-ment				% Overall agree-ment	% Overall agree-ment		% Overall agree-ment	% Overall agree-ment	
	Use the English word or fingerspell the word	44.8 (13/29)	53.8 (21/39)	65.0 (13/20)	0.462	0.585	0.245	66.7 (8/12)	62.5 (5/8)	1.000	75.0 (3/4)	62.5 (10/16)	1.000
	Ask the health service client to rephrase or explain	93.8 (30/32)	88.4 (38/43)	100 (22/22)	0.572	1.000	0.508	100.00 (13/13)	100.0 (9/9)	NC	100.0 (4/4)	100.0 (18/18)	NC
	Use a similar term or phrase	55.2 (16/29)	53.8 (21/39)	70.0 (14/20)	1.000	0.402	0.377	66.7 (8/12)	75.0 (6/8)	1.000	75.0 (3/4)	68.8 (11/16)	1.000

NC: not computable.
