# Supplementary material for: Evaluation of a Genetics Education Program for Health Interpreters: A Pilot Study
Source: Front Genet. 2022 Feb 3;12:771892. doi: 10.3389/fgene.2021.771892 (PMC8850313; doi:10.3389/fgene.2021.771892)
Supplement: Supplementary file 2 [file DataSheet1.PDF]

****Please note in this document words contained in [ ] are clarifying comments that was not present when surveys were entered into online platform. ****

**Information sheet for the pre-, post- and follow-up surveys**

# Evaluation of Medical Interpreter Training Session in Genomics

## INFORMATION SHEET FOR PARTICIPANTS

**Participation in this study is optional.**
**You can still attend the training session even if you do not participate in the study.** [pre-survey only]

**WHAT IS THE AIM OF THIS STUDY?**
We would like to find out if "Language of Genetics & Genomics: Healthcare Interpreter training session" was helpful for you in your work. In particular, we would like to know if it improves your knowledge, attitude and confidence about genetics and genomics.

**HOW WILL THE STUDY BE CARRIED OUT?**
There will be a series of three (3) online surveys. Each survey will take approximately 5-10 minutes to complete. The survey links will be sent to the email you registered with. They will be sent:

- Before the training session
- Just after the training session
- 6-months after the training session

**WILL ALL DATA PROVIDED BE CONFIDENTIAL?**
Yes. We will not collect any personal information and we will not be able to identify you from your survey answers. To link the survey responses we will use a self-generated code. Your survey responses need to be linked together and we will ask you to record a specific code on each survey. This code will only be known to you and will not identify you. We hope to publish the results of the survey in scientific journals and present the findings at conferences so that others can learn from this work. We will not report any individual results, only findings relating to the group of the participants. Data will be stored and archived in compliance with *NHMRC's Australian Code for the Responsible Conduct of Research*. Data access will be restricted to people named in this project.

**WHAT ARE THE RISKS AND BENEFITS OF PARTICIPATING IN THE STUDY?**
We do not anticipate any risks by taking part in this study. While there are no immediate benefits to you, your participation will help us to better understand how we can support interpreters during genetic and genomic consultations.

**WHAT IF I WISH TO WITHDRAW?**
**Participation in this study is voluntary**. You will not be able to withdraw your responses once you have completed a survey, but you can stop participating in the study by not completing future surveys that are emailed to you.

**WHO WILL BE INVOLVED IN THIS STUDY?**

| | |
|---|---|
| **Project Co-ordinator** | Dr. Miranda Vidgen, QIMR Berghofer |
| **Associated Investigators** | Dr. Nic Waddell, QIMR Berghofer |
| | Ms. Priya Ramarao-Milne, QIMR Berghofer |
| | Dr. Stephanie Best, Macquarie University & Australian Genomics |
| | Ms. Keri Finlay, University of Melbourne & Australian Genomics |
| | Dr. Lindsay Fowles, Genetic Health Queensland (Queensland Health) |
| | Dr. Erin Evans, Queensland Genomics |
| | Mr. Sartio Nindyo Isotiko, Queensland Genomics |

**IF YOU HAVE FURTHER QUESTIONS**
Please contact Miranda Vidgen via email: [email address supplied]

**LOW RISK APPROVAL:** QIMR Berghofer HREC project number P3471
This study has been approved by the QIMR Berghofer Medical Research institute Human Research Ethics Committee (HREC) as a low or negligible risk project. If you would like to speak with someone with regards to ethical matters or

wish to register a formal complaint about the conduct of this research - please contact the QIMR Berghofer HREC Secretary via email at [HREC email address supplied].

**Would you like to participate in this study? [Mandatory question]**

☐ Yes [goes to survey]

☐ No [goes to survey exit/thank you page]

## Core set of questions for the pre-, post- and follow-up surveys

**If you want to participate in the study please generate a unique code**

To compare change in knowledge and confidence over time, whilst also keeping your responses anonymous, we need you to create a unique code. In the box below enter the following in order:

1. First 3 letters of the month you were born (example: **<u>Mar</u>**ch)
2. Last two numbers of your mobile phone number (if you do not have a mobile phone number put 00) (example: 0491 570 1**<u>56</u>**)
3. Last two letters of the city you were born in (example: Brisba**<u>ne</u>**)

For the examples given above the code would be: MAR56NE

Please enter your unique code:

[Open text box]

**<u>Demographics</u>**

ITEM 1 **What is your age?**

☐ 18-24

☐ 25-34

☐ 35-44

☐ 45-54

☐ 55-64

☐ 65 and over

ITEM 2 **What is your gender?**

☐ Male

☐ Female

☐ Prefer to self-describe

ITEM 3 **Number of years working as a medical interpreter?**

☐ Not a medical interpreter

☐ Less than 1 year

☐ 1-5 years

☐ 6-10 years

☐ More than 10 years

ITEM 4 **Have you had any training in genetics?**

☐ None at all

☐ Some in high school or university

☐ Professional development or continued education

ITEM 5 **Have you interpreted for a specialist genetic clinician (clinical geneticist or genetic counsellor)?**

☐ Yes

☐ No

☐ Unsure

ITEM 6 **Have you interpreted genetic or genomic terms for a health service client before that is not a specialist genetic clinician (clinical geneticist or genetic counsellor)?**

☐ Yes
☐ No
☐ Unsure

ITEM 7 **Have you had personal experience outside your professional role (e.g. you, a friend or family member) with a significant genetic condition?**

☐ Yes
☐ No

ITEM 8 **What language(s) are you qualified to interpret?**

[Open text box]

**<u>Confidence related to interpreting genetic and genomic concepts [self-efficacy]</u>**

Rate how easy or difficult you find the following activities.

| | | Very difficult | Difficult | Neutral | Easy | Very Easy |
|---|---|---|---|---|---|---|
| ITEM 9 | Understanding genetic and genomic terms in English | ☐ | ☐ | ☐ | ☐ | ☐ |
| ITEM 10 | Explaining to others how genetics affects a person's health | ☐ | ☐ | ☐ | ☐ | ☐ |
| ITEM 11 | Interpreting information about genetics for my clients with limited English, or that are Deaf or hard of hearing | ☐ | ☐ | ☐ | ☐ | ☐ |

**<u>Perception of interpreting genetic and genomic concepts [attitude]</u>**

ITEM 12 **When compared to other medical services, how would you rate the importance of genetic health services to you and your family?**

[Slider scale from 1 to 5. Labels: 1 = Far Less, 3 = About the same & 5 = Far more]

ITEM 13 **When compared to other medical services, how would you rate the importance of genetic health services to your clients with limited English, or that are Deaf or hard of hearing?**

[Slider scale from 1 to 5. Labels: 1 = Far Less, 3 = About the same & 5 = Far more]

ITEM 14 **When compared to other medical terminology, how would you rate the importance of genetic and genomic terminology in you professional practice of interpreting?**

[Slider scale from 1 to 5. Labels: 1 = Far Less, 3 = About the same & 5 = Far more]

**<u>Behaviour related to interpreting genetic and genomic concepts [ self-reported practice behaviour]</u>**

Consider the following statements and indicate your level of agreement with the actions listed.

*In a client appointment if **<u>I do not know the word/sign for a genetic term</u>** in the language I am interpreting I….*

| | | Strongly Disagree | Disagree | Unsure | Agree | Strongly Agree |
|---|---|---|---|---|---|---|
| ITEM 15 | Use the English word or fingerspell the word | ☐ | ☐ | ☐ | ☐ | ☐ |
| ITEM 16 | Ask the health service client to rephrase or explain | ☐ | ☐ | ☐ | ☐ | ☐ |
| ITEM 17 | Use a similar term or phrase | ☐ | ☐ | ☐ | ☐ | ☐ |

*In a client appointment if* ***there is not an equivalent word/sign for a genetic term*** *in the language I am interpreting I….*

| | | Strongly Disagree | Disagree | Unsure | Agree | Strongly Agree |
|---|---|---|---|---|---|---|
| ITEM 18 | Use the English word or fingerspell the word | ☐ | ☐ | ☐ | ☐ | ☐ |
| ITME 19 | Ask the health service client to rephrase or explain | ☐ | ☐ | ☐ | ☐ | ☐ |
| ITEM 20 | Use a similar term or phrase | ☐ | ☐ | ☐ | ☐ | ☐ |

ITEM 21 **When interpreting for a client with limited English, or that are Deaf or hard of hearing, if there is not an equivalent word for a genetic or genomic term, what additional actions would you take to ensure their understanding of the term?**

[Open text box]

**Knowledge of genetic concepts**

Select if you think the statement is true or false.

*This is not a test of you as an individual. These questions will help determine what parts of the training session were effective for the group of people that participated.*

| | | I don't know | True | False |
|---|---|---|---|---|
| ITEM 22 | Some diseases are caused by genes, environment, and lifestyle. [True] | ☐ | ☐ | ☐ |
| ITEM 23 | You can see a gene with the naked eye. [False] | ☐ | ☐ | ☐ |
| ITEM 24 | Healthy parents can have a child with an inherited disease. [True] | ☐ | ☐ | ☐ |
| ITEM 25 | A gene is a piece of DNA. [True] | ☐ | ☐ | ☐ |
| ITEM 26 | A chromosome contains many genes. [True] | ☐ | ☐ | ☐ |
| ITEM 27 | Genes determine traits such as height, eye colour, and facial appearance. [True] | ☐ | ☐ | ☐ |
| ITEM 28 | A person has thousands of genes. [True] | ☐ | ☐ | ☐ |
| ITEM 29 | Humans have 24 pairs of chromosomes [False] | ☐ | ☐ | ☐ |
| ITEM 30 | Parents pass both copies of each chromosome to their child. [False] | ☐ | ☐ | ☐ |
| ITEM 31 | A genetic test can tell you if you have a higher chance to develop a specific disease. [True] | ☐ | ☐ | ☐ |

## Extra questions in the post training survey

**Training session feedback**

With regard to this training session, indicate your level of agreement with the following statements.

| | | Strongly Disagree | Disagree | Unsure | Agree | Strongly Agree |
|---|---|---|---|---|---|---|
| ITEM 1 | The content was clearly presented | ☐ | ☐ | ☐ | ☐ | ☐ |
| ITME 2 | The content was informative | ☐ | ☐ | ☐ | ☐ | ☐ |
| ITEM 3 | The group activities improved my learning | ☐ | ☐ | ☐ | ☐ | ☐ |
| ITEM 4 | The case study activity improved my learning | ☐ | ☐ | ☐ | ☐ | ☐ |
| ITEM 5 | The online delivery format was easy to use | ☐ | ☐ | ☐ | ☐ | ☐ |
| ITEM 6 | The online delivery format was engaging | ☐ | ☐ | ☐ | ☐ | ☐ |
| ITEM 7 | The training session was useful for my work | ☐ | ☐ | ☐ | ☐ | ☐ |
| ITEM 8 | The training session was relevant to my work | ☐ | ☐ | ☐ | ☐ | ☐ |

ITEM 9 **Do you have any further suggestions about how to improve this training session?**

[Open text box]

ITEM 10 **Which part of the training session did you find most valuable/enjoyable?**

[Open text box]

## Extra questions in the 6-month follow-up survey

**Clinical experience**

ITEM 1 **Since completing the training session have you done any additional learning about genetics and genomics?**

- ☐ No
- ☐ Yes, looked at materials provided from training session
- ☐ Yes, looked at other materials not provided in training session
- ☐ Yes, looked at both

ITEM 2 **Since completing the training session have you had a client appointment where you interpreted genetics and genomics terms?**

- ☐ Yes [survey goes to Continue Clinical experience]
- ☐ No [survey skips Continue Clinical experience]

**Continue Clinical experience**

When answering the following questions think about client appointments that have occurred **since** the training session where you have interpreted genetic or genomic terms.

ITEM 3 **Since completing the training session, in how many appointments have you interpreted genetic terms?**

- ☐ 1-3
- ☐ 4-6
- ☐ >6

ITEM 4 **Since completing the training session, how many times did you interpret genetic terms for these medical specialities?**

[Each specialty has a dropdown box with options: 0, 1, 2 more than 3]

- Specialist genetic clinician (clinical geneticist or genetic counsellor)
- Allergies and Immunology
- Breast and Endocrine surgery
- Cardiology
- Endocrinology
- General Practice
- Gynaecology
- Maternity and neonatal medicine
- Neurology
- Oncology
- Paediatric
- Renal medicine (Nephrology)

ITEM 5 **Think about client appointments that have occurred since the training session where you have interpreted genetic or genomic terms. How often were these appointments conducted in the following settings?**

[Each setting has a dropdown box with options: 0, 1, 2 more than 3]

- Face to Face (interpreter and clients all in clinical setting)
- Telehealth (interpreter and clients using teleconferencing technology)
- Telephone (interpreter and clients all using telephones)
- Teleconferencing or telephone by interpreter only (interpreter using teleconferencing or telephone and clients in face to face clinical setting)
