# Supplementary figures and images for: Evaluation of a Genetics Education Program for Health Interpreters: A Pilot Study
Source: Front Genet. 2022 Feb 3;12:771892. doi: 10.3389/fgene.2021.771892 (PMC8850313; doi:10.3389/fgene.2021.771892)

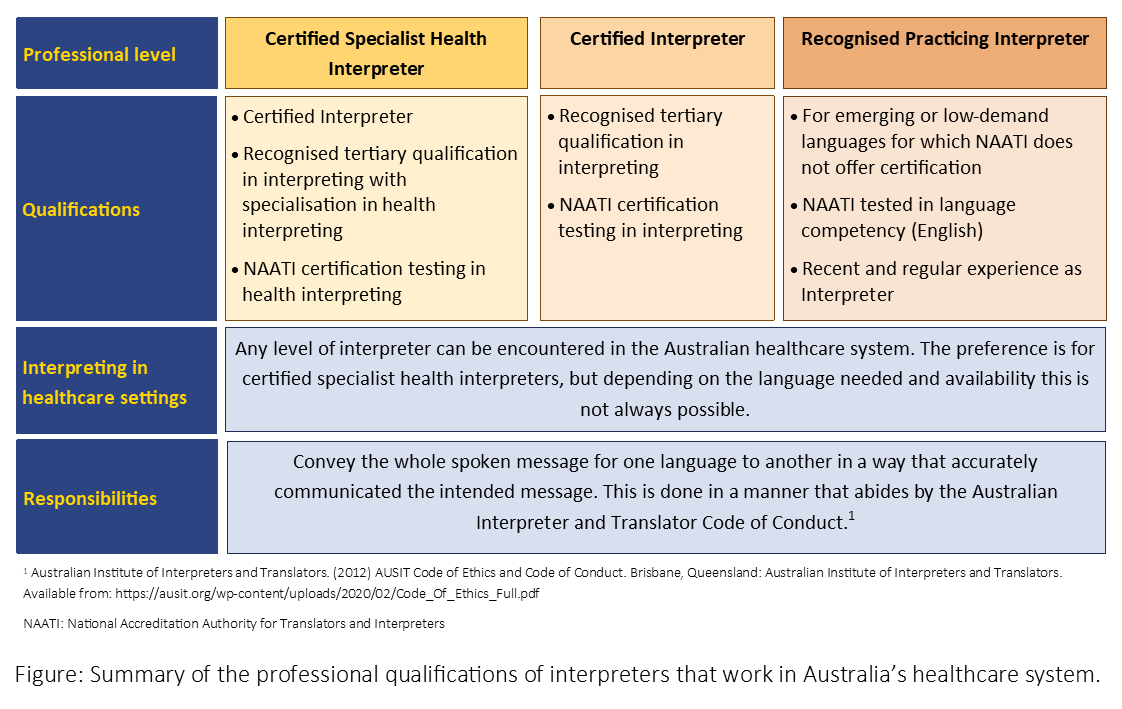

Supplement: Supplementary file 3 [file Image1.png]
